# Dynamic Sleep-Derived Heart Rate and Heart Rate Variability Features Associated with Glucose Metabolism Status: An Exploratory Feature-Selection Study Using Consumer Wearables

**DOI:** 10.3390/s26041118

**Published:** 2026-02-09

**Authors:** Li Li, Syarifah Nabilah Syed Taha, Yoshiyuki Nishinaka, Yufeng Tan, Hajime Ohtsu, Sinyoung Lee, Ken Kiyono

**Affiliations:** 1 Graduate School of Engineering Science, The University of Osaka, Osaka 560-8531, Japan; 2 IntaSect Communications, Inc., 3-1 Kanda Ogawamachi, Chiyoda-ku, Tokyo 101-0052, Japan

**Keywords:** wearable sensors, heart rate variability (HRV), glucose metabolism, elastic net regression, sleep physiology, autonomic function

## Abstract

Impaired glucose metabolism, a known precursor to type 2 diabetes, is associated with dysregulation of the autonomic nervous system. To assess such autonomic states, consumer wearable devices provide continuous, non-invasive physiological monitoring and may capture autonomic signatures related to metabolic status. This exploratory study examined whether dynamic features of heart rate (HR) and heart rate variability (HRV) during sleep—derived from a consumer wrist-worn device (Fitbit)—are associated with glucose metabolism status in free-living adults. We analyzed 189 nights from 18 participants (7 participants in the higher-glycemic-risk group, estimated glycated hemoglobin (HbA1c) ≥ 5.5%; 11 participants in the lower-glycemic-risk group, estimated HbA1c < 5.5%). From 28 candidate HR/HRV variables, Elastic Net regression (α=0.5) was applied to identify features associated with nocturnal mean glucose. Fourteen features retained non-zero coefficients; notably, dynamic features capturing overnight trends and variability patterns showed stronger associations than conventional static mean values. The nocturnal trends of within-window standard deviation and variance of ln(RMSSD) (root mean square of successive differences between consecutive RR intervals, estimated here from PPG-derived inter-beat intervals; RMSSD) emerged as prominent candidates, alongside HR variability indices. Independent between-group comparisons further confirmed that two dynamic HRV features differed significantly between the lower- and higher-glycemic-risk groups (both p<0.05; Cohen’s |d|>1.1). Specifically, the lower-glycemic-risk group exhibited decreasing overnight trends in HRV variability, consistent with progressive autonomic stabilization during sleep. In contrast, the higher-glycemic-risk group showed increasing variability trends, suggestive of persistent autonomic instability. These directional patterns are consistent with prior evidence linking autonomic dysfunction to impaired glucose metabolism. We characterize these findings as hypothesis-generating. The identified dynamic HR/HRV features represent physiologically plausible candidate correlates of glycemic status and warrant confirmatory investigation in larger, independent cohorts with laboratory-measured HbA1c. More broadly, this work highlights the potential of widely available, consumer-grade wearable devices to move beyond activity tracking and support continuous, real-world assessment of cardiometabolic health, thereby expanding their utility in everyday health monitoring and preventive medicine.

## 1. Introduction

Diabetes mellitus and prediabetes remain major global public health challenges. The International Diabetes Federation’s (IDF) Diabetes Atlas (11th edition) estimates that 11.1% of adults aged 20–79 years (approximately 589 million people) had diabetes in 2025, and projects an increase to about 853 million by 2050 (approximately 12.5% of adults) [1]. Importantly, more than 40% of adults with diabetes are estimated to be undiagnosed, highlighting persistent gaps in early detection of impaired glucose metabolism [1]. From the perspective of primary prevention of type 2 diabetes, identification at the prediabetes stage is particularly important. In the Diabetes Prevention Program (DPP), intensive lifestyle intervention and metformin reduced the risk of developing type 2 diabetes by 58% and 31%, respectively, compared with placebo among individuals with impaired glucose tolerance [2]. These findings underscore the importance of identifying high-risk individuals at early stages and linking them to effective interventions.

Fasting-plasma glucose (FPG) and glycated hemoglobin (HbA1c) are widely used for screening impaired glucose metabolism, but both require invasive blood sampling and are typically obtained at discrete time points in clinical practice, which may present practical challenges for frequent, large-scale primary screening outside clinical settings.

Moreover, HbA1c reflects average glycemic exposure over the preceding 2–3 months and does not capture day-to-day glycemic variability (GV). Previous studies have highlighted the clinical relevance of GV beyond mean glucose levels; Suh and Kim summarized common GV metrics, such as SD, MAGE, and CV, and discussed associations with oxidative stress and endothelial dysfunction [3], while Monnier et al. reported that acute glucose fluctuations were more strongly associated with oxidative stress markers than sustained hyperglycemia [4]. Although continuous glucose monitoring (CGM) enables detailed assessment of glycemic profiles, including GV, it requires subcutaneous sensor placement and remains less suitable for broadly accessible, low-cost primary screening. These limitations motivate the search for noninvasive indicators that can be deployed at scale.

Autonomic nervous system (ANS) function has emerged as a potential target for the early identification of metabolic risk. Glucose homeostasis is regulated in part by the balance between sympathetic and parasympathetic activity, and autonomic imbalance has been implicated in insulin resistance and the pathophysiology of type 2 diabetes. Population-based studies suggest that autonomic dysfunction may function not merely as a downstream consequence of diabetes but also as an aggravating factor or a parallel pathological process. For example, the Toon Health Study reported that reduced heart rate variability (HRV) and relative sympathetic dominance modified the association between insulin resistance and metabolic syndrome [5].

Cardiac autonomic neuropathy (CAN) has also been documented in individuals with prediabetes, with reported prevalence estimates ranging from 9 to 38%. Moreover, large cohort studies, such as the Cooperative Health Research in the Region of Augsburg (KORA) S4, have demonstrated higher rates of CAN in prediabetes compared with normal glucose tolerance [6,7]. Consistent with these findings, recent reviews have further supported the notion that autonomic dysfunction may precede the clinical onset of diabetes [8].

Heart rate variability (HRV) is widely used as a noninvasive surrogate marker of autonomic function, with established physiological interpretations described in the Task Force guideline and subsequent reviews [9,10]. Epidemiological evidence consistently links reduced HRV to adverse glucose metabolism. Meta-analyses have reported lower HRV across multiple indices in type 2 diabetes and metabolic syndrome, including SDNN (standard deviation of normal-to-normal intervals) and RMSSD (root mean square of successive differences between consecutive RR intervals, estimated here from PPG-derived inter-beat intervals), total power, and HF power (high-frequency power, typically 0.15–0.40 Hz) [11,12]. Population-based studies, such as the Maastricht Study, have further shown that HRV indices are reduced not only in type 2 diabetes but also in prediabetes compared with normal glucose metabolism [13]. Longitudinal studies suggest that temporal patterns of HRV provide additional prognostic information; for example, HRV trajectories predicted diabetes onset in the Rotterdam Study [14], and multiple HRV indices independently predicted diabetes risk in ELSA-Brasil [15]. These findings motivate attention to dynamic autonomic patterns rather than single timepoint summaries.

Associations between autonomic function and glucose metabolism may be particularly evident during sleep, when major daytime confounders such as physical activity, diet, and acute psychological stress are minimized. Experimental studies have demonstrated that selective slow-wave sleep suppression reduces insulin sensitivity by approximately 25% [16], and that sleep fragmentation worsens glucose metabolism via sympathetic activation [17]. Epidemiological evidence further links sleep characteristics with glycemic markers and diabetes risk, including associations between poor sleep quality and higher HbA1c [18], and U-shaped relationships between sleep duration and diabetes risk [19].

Importantly, a study using 24-h ECG recordings in patients with type 2 diabetes demonstrated that sleep-stage-specific HRV indices—particularly nonlinear measures analyzed separately for REM and NREM sleep—were significantly associated with metabolic function and glycemic control [20].

Together, these observations provide physiological and methodological support for focusing on sleep-time HR and HRV dynamics.

Recent advances in consumer wearable sensors enable longitudinal monitoring of physiological signals such as heart rate (HR) and HRV in daily life. Large-scale studies have linked wearable-derived data to chronic disease outcomes, including type 2 diabetes [21,22]. Although pulse rate variability (PRV) derived from photoplethysmography (PPG) does not perfectly match ECG-derived HRV, validation studies support PPG-based indices as practical surrogates under appropriate conditions. Agreement between PRV and HRV has been reported at rest and during free-living conditions [23,24], with particularly high accuracy during sleep when motion artifacts are minimized [25,26]. These findings support the feasibility of sleep-time wearable monitoring as a noninvasive approach to assessing autonomic dynamics related to glycemic status.

The primary objective of this exploratory study is to identify sleep-derived HR/HRV features—particularly dynamic features capturing variability, trends, and instability—that are associated with glucose metabolism status. We hypothesize that dynamic autonomic indices, such as overnight trends in HRV dispersion and patterns of HR variability, show stronger associations with glycemic markers than conventional static mean values. To address this objective, we apply LASSO (Least Absolute Shrinkage and Selection Operator)-based feature selection to screen candidate features and conduct between-group comparisons to assess effect sizes, with the aim of generating hypotheses for confirmatory investigation in larger cohorts.

## 2. Materials and Methods

### 2.1. Participants and Study Design

This prospective observational study enrolled adults who wore continuous glucose-monitoring (CGM) devices and wrist-worn wearable devices simultaneously under free-living conditions. Twenty-one adults were initially enrolled in the study. Three participants were excluded due to incomplete data (insufficient valid sleep-time HRV recordings or CGM data). The final analytical sample comprised 18 participants with valid sleep-time heart rate variability (HRV) data (9 males and 9 females; mean age: 39.6 ± 10.4 years; mean body mass index (BMI): 22.7 ± 2.3 kg/m^2^) were included in the analysis. Based on preliminary screening, participants had no confirmed diagnosis of diabetes, no history of serious cardiovascular disease (e.g., heart failure, severe arrhythmia, ischemic heart disease), and no skin conditions or allergies that would interfere with device wear.

This study was approved by the Institutional Review Board of The University of Osaka (protocol code R4-17).

Before participation, all participants received oral and written explanations of the study objectives, procedures, anticipated risks and benefits, and privacy protections. Participation was voluntary, and participants could withdraw at any time without disadvantage. Written informed consent was obtained from all participants. Data were managed using anonymized research IDs and analyzed and reported in a non-identifiable form.

Participants were instructed to wear the devices continuously for up to 15 days during daily life. Day 1 served as an acclimation period; data collected from Day 2 onward were used for analysis.

Continuous Glucose Monitoring: Abbott FreeStyle Libre sensors were worn on the upper arm to record interstitial glucose continuously at approximately 15 min intervals. CGM systems, including FreeStyle Libre, measure interstitial glucose via electrodes and transmit values to receivers or smartphones at intervals ranging from several minutes to 15 min [27,28,29]. Glucose values with timestamps were extracted using the pipeline described below and used to compute daily mean glucose and the glucose management indicator (GMI; estimated HbA1c).

Wearable Device (Heart Rate, Activity, and HRV): Heart rate (HR) and HRV were measured using Fitbit Charge 6 wrist-worn activity trackers (Google LLC, Mountain View, CA, USA). The device employs an optical heart rate sensor based on wrist reflectance photoplethysmography (PPG), for which green light (wavelength approximately 525 nm) is commonly used in consumer wearables to achieve favorable signal quality at the wrist while reducing susceptibility to motion artifacts compared with longer-wavelength light sources, such as red or infrared LEDs [30,31,32,33].

The device internally samples the PPG signal at a frequency sufficient for beat-to-beat interval detection. For research analysis, the data are downsampled and aggregated: heart rate values are recorded at 1-s intervals during exercise and 5-s intervals during standard monitoring (including sleep). HRV metrics (specifically RMSSD) are computed by the device over non-overlapping 5-min windows based on high-resolution beat-to-beat intervals and are reported as discrete values at 5-min intervals [33,34]. Sleep period detection was performed using the Fitbit device’s proprietary sleep-staging algorithm. Although the specific algorithm implemented in the Charge 6 has not been independently validated in peer-reviewed literature, previous validation studies of earlier Fitbit models with sleep-staging capabilities against polysomnography have reported high sensitivity (0.95–0.96) for detecting sleep epochs [34].

Sleep periods were defined using sleep onset and wake time estimates (StartTime and EndTime) provided by the device. For each participant and night, all physiological time-series data (HR and HRV) were temporally aligned, and only data points falling within the interval between the estimated sleep onset and wake time were extracted and analyzed as sleep-period data. No sleep-stage-specific classification was applied; sleep periods were treated in a stage-agnostic manner based solely on the device-estimated sleep onset and offset.

### 2.2. Feature Extraction: Two-Stage Approach

Features were extracted using a two-stage hierarchical approach: (1) window-level statistics computed within each 30 min sleep window, and (2) night-level aggregation across all windows within a sleep session.

#### 2.2.1. Stage 1: Window-Level Feature Extraction

Sleep periods were segmented into non-overlapping 30 min windows. For each window *w*, we computed descriptive statistics for heart rate (HR) and log-transformed RMSSD (ln RMSSD) based on the time series *x* sampled within that window.

Within-Window Descriptors

For each 30 min window *w*, let *x* denote the subwindow time series consisting of all HR or (ln RMSSD) samples observed within the window. The following within-window descriptors were computed from this subwindow time series: the mean, defined as the average value of *x* within the window; the standard deviation, defined as the dispersion of *x* around its window-specific mean; and the variance, defined as the squared standard deviation, reflecting the overall variability of *x* within the window.

Within-Window Linear Slope

To characterize short-term monotonic trends, we fitted a linear model to the subwindow time series *x* within each 30 min window *w*:

(1) $$x_{i}=\beta_{0,w}+\beta_{1,w}\;t_{i}^{\left(\mathrm{hr}\right)}+\varepsilon_{i},$$

where $t_{i}^{\left(\mathrm{hr}\right)}$ denotes the elapsed time in hours from the start of window *w* (range: 0–0.5 h). The parameters are defined as follows:

- $\beta_{1,w}$ (Slope): This represents the rate of change in the physiological metric *x* per hour, quantifying the direction and magnitude of the monotonic trend within the window.
- $\beta_{0,w}$ (Intercept): This parameter represents the fitted baseline value of the metric at the exact start of the window (t=0). Physiologically, this estimates the initial level of the autonomic parameter for that specific epoch, filtering out the subsequent temporal drift.
- $\varepsilon_{i}$ (Residual): Residual represents the deviation of the observed data point from the fitted linear trend at time *i*. This term captures higher-frequency fluctuations and short-term variability (such as instantaneous autonomic responses to micro-arousals) not explained by the linear trajectory.

The estimated slope $\beta_{1,w}$ has units of change per hour (e.g., bpm/h for HR and ln(ms)/h for ln(RMSSD)). Here, bpm/h (beats per minute per hour) denotes the change in heart rate (measured in beats per minute) per hour of elapsed time. For example, $\beta_{1,w}=2$ bpm/h means that HR increases by 2 bpm for every hour of elapsed time within the 30 min window. Similarly, ln(ms)/h denotes the change in ln(RMSSD) per hour of elapsed time.

Within-Window Speed

As a complementary, nonparametric measure of trend magnitude, we computed the endpoint-to-endpoint rate of change within each window as

(2) $$v_{w}=\frac{x\left(t_{w}+30\;\min\right)-x\left(t_{w}\right)}{30\;\min},$$

where $t_{w}$ denotes the start time of window *w*. Accordingly, $v_{w}$ has units per minute (e.g., bpm/min for HR).

#### 2.2.2. Stage 2: Night-Level Aggregation

For each participant-night *d*, multiple valid non-overlapping 30 min sleep windows were retained. Let $W_{d}$ denote the number of retained windows for night *d*, and let $z_{d,w}$ denote a window-level descriptor computed from heart rate (HR) or natural-log-transformed RMSSD (lnRMSSD) in window $w\in W_{d}$.

Window-level descriptors were aggregated across windows to construct night-level features that characterize the overall level, temporal evolution, and across-window variability of autonomic dynamics during the sleep session.

Nightly Summary Statistics

To summarize the overall level and across-window variability of a window-level descriptor $z_{d,w}$ during a sleep session, we computed the nightly mean and the nightly standard deviation across all retained windows within night *d*. The nightly mean represents the typical level of the descriptor over the night, whereas the nightly standard deviation quantifies window-to-window variability. The latter was primarily used for dynamic window-level descriptors, such as within-window linear slopes and endpoint-to-endpoint speeds, which explicitly reflect short-term fluctuations within each 30 min window.

Nocturnal Trend

To capture systematic changes in window-level descriptors over the course of sleep, we regressed $z_{d,w}$ on elapsed time from the first retained window of the night:

(3) $$z_{d,w}=a_{d}+b_{d}\;\Delta t_{d,w}+\varepsilon_{d,w},$$

where $\Delta t_{d,w}$ denotes the elapsed time in minutes from the start of the first retained window of night *d*.

The slope $b_{d}$, referred to as the nocturnal trend, quantifies whether the window-level descriptor systematically increases or decreases over the night. The nocturnal trend has units per minute (e.g., bpm/min for HR, ln(ms)/min for ln(RMSSD)) because $\Delta t_{d,w}$ is expressed in minutes. This differs from the within-window slopes $\beta_{1,w}$, which use elapsed time in hours and thus have units per hour.

Night-Level Features Used for LASSO Screening

For LASSO-based feature screening, night-level features were constructed as follows: For window-level descriptors reflecting distributional properties within each 30 min window (window-wise mean, standard deviation, variance, minimum, and maximum of HR and lnRMSSD), we used the nightly mean and the nocturnal trend. For dynamic window-level descriptors characterizing short-term changes within each window (within-window linear slope and endpoint-to-endpoint speed), we used the nightly mean and the nightly standard deviation across windows.

A one-to-one correspondence between these manuscript definitions and the feature identifiers used in the analysis is provided in Supplementary Table S1.

### 2.3. Glucose Metabolism Status Definition and Outcome Labeling

#### 2.3.1. Estimated HbA1c (eHbA1c) from CGM Mean Glucose

For each participant, we computed an HbA1c-equivalent index from the participant-level mean CGM glucose (mg/dL) using the A1c-Derived Average Glucose (ADAG) relationship:

(4) $$\mathrm{eHbA}1c\left(\%\right)=\frac{\mathrm{mean}\;\mathrm{glucose}\;(\mathrm{mg}/\mathrm{dL})+46.7}{28.7}.$$

This expression is a rearrangement of the ADAG regression linking laboratory-measured HbA1c to average glucose, providing an HbA1c-equivalent summary derived from mean glucose [35].

The glucose management indicator (GMI) has been proposed as an alternative CGM-based index that expresses mean glucose on an HbA1c-like scale using a CGM-specific regression [36]. Because eHbA1c/GMI may differ from laboratory HbA1c due to inter-individual factors (e.g., variability in erythrocyte lifespan and glycation kinetics), such CGM-derived indices should be interpreted as HbA1c-equivalent proxies rather than diagnostic measurements [37].

#### 2.3.2. Outcome Labeling for Machine Learning

We defined a screening-oriented binary outcome at the participant level using a CGM-derived eHbA1c threshold of 5.5%. Participants with eHbA1c < 5.5% were classified as the lower-glycemic-risk group, whereas those with eHbA1c ≥ 5.5% were classified as the higher-glycemic-risk group. This threshold was selected for risk stratification based on evidence from Japanese cohorts evaluating HbA1c cutoffs for future type 2 diabetes risk (Hisayama Study) [38] and supported by longitudinal findings reporting substantially elevated diabetes incidence within the HbA1c 5.7–6.4% range (TOPICS 3) [39]. We emphasize that these group labels are derived from a CGM-based HbA1c-equivalent proxy and are used solely to operationalize an outcome for screening-oriented research; they do not constitute a clinical diagnosis.

We selected 5.5% as a conservative, sensitivity-prioritized cutoff for early-stage screening in an apparently healthy population based on the following considerations.

1. **Japanese epidemiological evidence.** In the Hisayama Study, a population-based prospective cohort of Japanese adults (N=2016; 7-year follow-up), individuals with baseline HbA1c 5.5–5.9% had approximately a 4.8-fold higher risk of developing type 2 diabetes than those with HbA1c < 5.5% (95% CI: 2.0–11.6) [38]. Similarly, the Toranomon Hospital Health Management Center study (TOPICS 3; N=6241) reported substantially elevated annual diabetes incidence in the 5.7–6.4% range [39].
2. **Asian-specific risk profiles.** Meta-analyses suggest that Asian populations may exhibit increased diabetes risk at lower HbA1c levels than Western populations, potentially due to differences in beta-cell function and insulin sensitivity [40]. This supports adopting a lower screening threshold in Japanese cohorts.
3. **Screening philosophy.** For primary screening aimed at identifying individuals who may benefit from early lifestyle intervention, prioritizing sensitivity is methodologically appropriate. The 5.5% cutoff may help detect early glycemic dysregulation before progression to the ADA-defined prediabetes range (5.7–6.4%) or overt diabetes (≥6.5%).

We acknowledge that this threshold is lower than the ADA prediabetes criterion (5.7%) [41]. Therefore, individuals identified as screen-positive by the model would require confirmatory laboratory testing (measured HbA1c and/or OGTT) before clinical classification.

### 2.4. Feature Selection: LASSO-Based Variable Screening

The primary analytical objective was to identify candidate HR/HRV features associated with glucose metabolism indicators.

Outcome Variable

Nocturnal mean glucose (continuous) at the participant–night level was used as the outcome variable for feature screening.

Candidate Predictors

Candidate predictors included 28 HR/HRV-derived variables together with demographic covariates (age and BMI). Variables with more than 50% missing values or zero variance were excluded. The remaining values were imputed using a two-stage procedure: within-subject mean imputation followed by overall mean imputation. All predictors were standardized to have zero mean and unit standard deviation prior to model fitting.

Elastic Net Regression

Elastic Net regression was applied with α=0.5, corresponding to equal weighting of LASSO and ridge penalties. LASSO is a regularized regression technique that adds an L_1_ penalty term ($\lambda \sum |\beta_{j}|$) to the standard least squares objective function. This penalty shrinks the coefficients of less informative predictors toward zero and, critically, forces some to become exactly zero, thereby performing automatic feature selection. However, when predictors are highly correlated (as HR/HRV features often are due to shared physiological origins), LASSO may arbitrarily select one variable and ignore others. Elastic Net addresses this by combining the L_1_ penalty with the L_2_ penalty of Ridge regression ($\lambda \sum \beta_{j}^{2}$), providing both sparsity (feature selection) and robustness to multicollinearity [42,43]. The regularization parameter λ was selected using 5-fold cross-validation. Features with non-zero coefficients at the optimal value of λ were retained as candidate correlates of glucose metabolism.

### 2.5. Between-Group Comparisons

Independent of the feature screening stage, we conducted univariate between-group comparisons at the participant level to assess whether individual HR/HRV features differed between the lower- and higher-glycemic-risk groups. Because group sizes were unequal and homogeneity of variance could not be assumed, Welch’s *t*-test was used for continuous variables. Effect sizes were quantified using Cohen’s *d* to complement p-values and to facilitate interpretation of the magnitude and practical relevance of between-group differences.

### 2.6. Statistical Analysis

All analyses were performed in MATLAB R2025a. Baseline demographic characteristics were compared between groups using Welch’s *t*-test for continuous variables and Fisher’s exact test for categorical variables, as appropriate. Statistical significance was assessed at a two-sided threshold of p<0.05.

Given the exploratory nature of this study and the limited sample size, we report unadjusted *p*-values and emphasize effect sizes and consistency of findings across related features. Accordingly, statistical results should be interpreted as hypothesis-generating rather than confirmatory, and any potentially informative signals identified here warrant validation in larger, independent cohorts.

## 3. Results

This section first reports the results obtained using the dynamic autonomic indices newly introduced in this study. These indices, derived from sleep-period heart rate (HR) and heart rate variability (HRV) signals—such as HR change speed and slopes of HRV-related measures—were designed to capture temporal trends and fluctuations in autonomic regulation during sleep. We found that these dynamic indices showed stronger associations with individual glucose metabolism status than conventionally used static mean HR/HRV indices.

This section then presents participant characteristics, results of feature selection, classification performance, and between-group comparisons. Overall, the dynamic autonomic indices, which reflect time-varying sympathetic and parasympathetic activity, demonstrated higher explanatory power for nocturnal mean glucose than static summary measures, supporting their potential utility for screening-oriented assessment of glycemic risk.

### 3.1. Participant Characteristics

The analytical sample comprised 18 participants contributing 189 person–nights. In total, 7 participants (38.9%) were classified as the higher-glycemic-risk group (eHbA1c ≥ 5.5%), and 11 participants (61.1%) as the lower-glycemic-risk group (eHbA1c < 5.5%). Age and BMI did not differ significantly between groups (both p>0.30).

**Figure 1:**
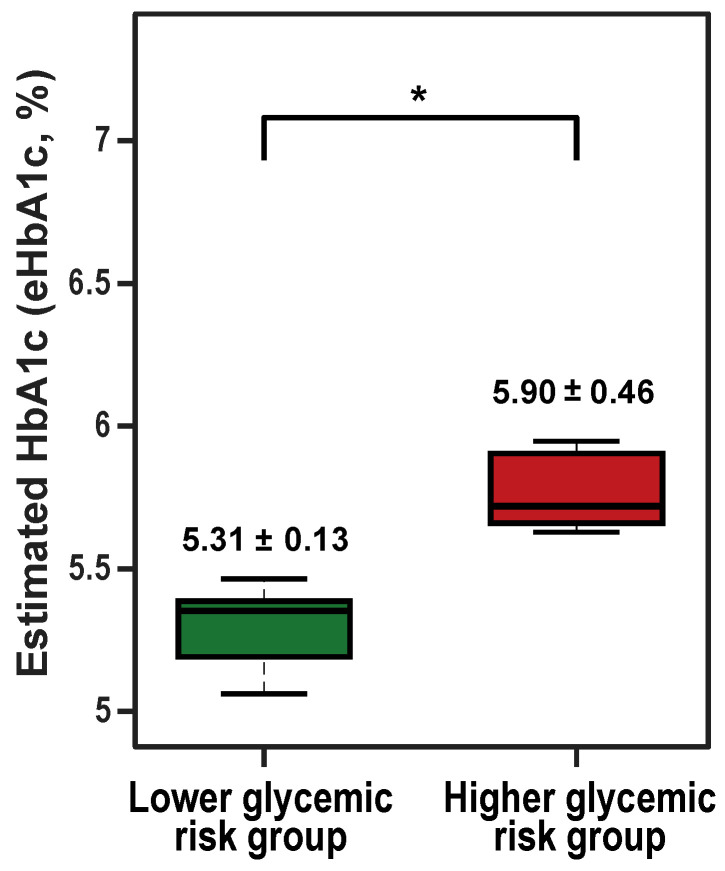
Between-group comparison of estimated HbA1c (eHbA1c) by glycemic status. eHbA1c was derived from continuous glucose monitoring (CGM) data using the glucose management indicator (GMI) formula. Participants were classified into the lower-glycemic-risk group (eHbA1c < 5.5%; n=11; mean ± SD: 5.31±0.13%) and the higher-glycemic-risk group (eHbA1c ≥ 5.5%; n=7; mean ± SD: 5.90±0.46%). Values above the boxes indicate mean ± SD. Box boundaries represent the interquartile range (IQR; 25th–75th percentiles), the center line indicates the median, and whiskers extend to 1.5 × IQR. Group differences were assessed using Welch’s *t*-test; * p<0.05.

The mean glucose and eHbA1c were significantly higher in the higher-glycemic-risk group (both p<0.001; Table 1). Figure 1 shows the between-group comparison of eHbA1c by glycemic status.

### 3.2. Feature Selection Results: Dynamic Features Show Stronger Associations

**Figure 2:**
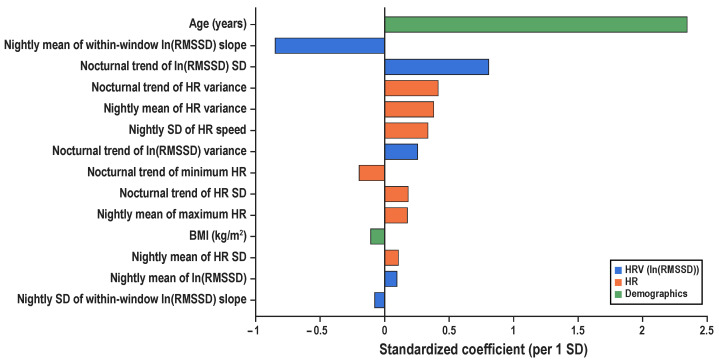
Standardized LASSO coefficients from Stage-1 feature screening. Elastic Net regression (α=0.5) with 5-fold cross-validation was applied to identify candidate features associated with nocturnal mean glucose (continuous outcome). Coefficients are expressed per 1-SD increase in each predictor, allowing for direct comparison of relative effect sizes across features. Only features with non-zero coefficients are shown. Color coding indicates feature categories: blue = HRV-derived features based on ln(RMSSD); orange = HR-derived features; green = demographic covariates. Among the 14 features retained at this stage, the top eight were selected as primary candidates for subsequent analyses.

Elastic Net regression (α=0.5; 5-fold CV) identified 14 features with non-zero coefficients from 28 initial HR/HRV variables plus demographics (Figure 2).

#### 3.2.1. Demographic Covariates

Age exhibited the largest standardized coefficient ($\beta_{\mathrm{std}}\approx 2.3$), indicating a strong positive association with nocturnal mean glucose. BMI also showed a positive contribution. These associations are consistent with established epidemiological evidence linking older age and higher BMI to impaired glucose metabolism.

#### 3.2.2. HRV Dynamic Indices

Among the dynamic features derived from ln(RMSSD), three indices retained non-zero coefficients in the feature selection procedure.

The first was the nightly mean of the within-window linear slopes, denoted as $\bar{\beta_{1,w}^{\left(\ln(\mathrm{RMSSD})\right)}}$. This index represents the average linear slope of ln(RMSSD) computed within individual 30 min sleep windows and then averaged across all windows within a night. As such, it summarizes the typical short-term change in parasympathetic activity during sleep, with positive values indicating that ln(RMSSD) tended to increase within windows on average.

The second index was the nocturnal trend in the within-window standard deviation, $b_{\sigma }^{\left(\ln(\mathrm{RMSSD})\right)}$. This feature was obtained by regressing the window-level standard deviation of ln(RMSSD), $\sigma_{w}^{\left(\ln(\mathrm{RMSSD})\right)}$, on elapsed time across the night (Equation (3)). It captures whether HRV variability systematically increases (b>0) or decreases (b<0) as sleep progresses.

The third index, $b_{s^{2}}^{\left(\ln(\mathrm{RMSSD})\right)}$, represents the nocturnal trend of the within-window variance of ln(RMSSD). Analogous to the standard-deviation-based index, this measure was computed using the window-level variance ${\left(s_{w}^{2}\right)}^{\left(\ln(\mathrm{RMSSD})\right)}$ and reflects changes in the overall dispersion of parasympathetic activity over the course of the night.

#### 3.2.3. HR Dynamic Indices

In addition to HRV-based measures, three dynamic features derived from heart rate (HR) also retained non-zero coefficients in the feature selection procedure.

One such feature was the nocturnal trend of the within-window HR variance, denoted as $b_{s^{2}}^{\left(\mathrm{HR}\right)}$. This index was obtained by regressing the window-level HR variance, ${\left(s_{w}^{2}\right)}^{\left(\mathrm{HR}\right)}$, on elapsed time across the night. It captures whether short-term HR dispersion systematically increases (b>0) or decreases (b<0) as sleep progresses.

Another retained feature was the nightly mean of the within-window HR variance, $\bar{{\left(s_{w}^{2}\right)}^{\left(\mathrm{HR}\right)}}$, which reflects the average magnitude of short-term HR variability across all 30 min sleep windows within a night. This measure summarizes the overall level of HR instability during sleep.

The third feature was the nightly standard deviation of the within-window HR speed, $\mathrm{SD}\;\left(v_{w}^{\left(\mathrm{HR}\right)}\right)$. This index quantifies how variable the rate of HR change is from window to window over the course of the night, with higher values indicating more heterogeneous dynamics, characterized by rapid HR changes in some windows and minimal changes in others.

### 3.3. Between-Group Differences in Dynamic Features

**Figure 3:**
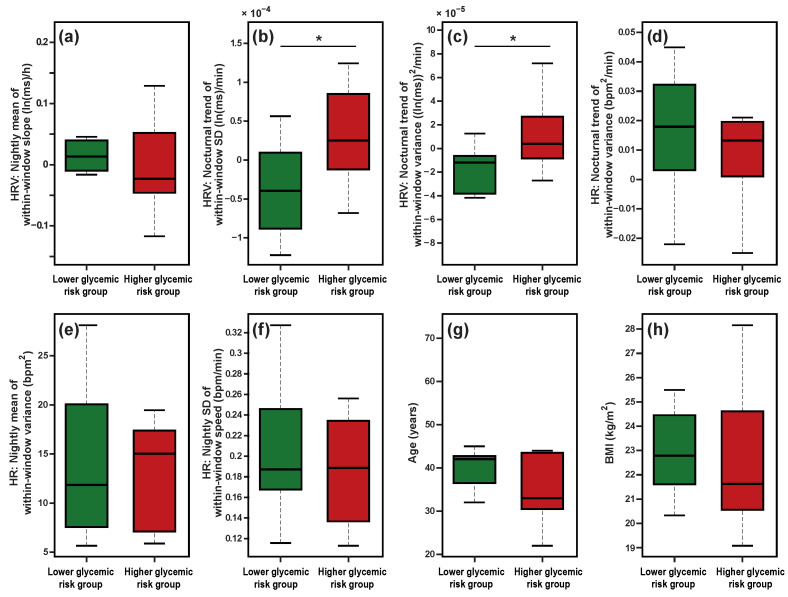
Subject-level comparison of candidate HR and HRV features between the lower-glycemic-risk group (eHbA1c < 5.5%; n=11) and the higher-glycemic-risk group (eHbA1c ≥ 5.5%; n=7). Each boxplot displays the distribution of participant-level mean values (one value per participant, aggregated across all valid sleep nights). Group differences were evaluated using Welch’s *t*-test at the subject level, and effect sizes are reported as Cohen’s *d*. Two HRV-related indices—the nocturnal trend of within-window SD ($b_{\sigma }^{\left(\ln\mathrm{RMSSD}\right)}$; panel (**b**)) and the nocturnal trend of within-window variance ($b_{\sigma^{2}}^{\left(\ln\mathrm{RMSSD}\right)}$; panel (**c**))—showed statistically significant between-group differences (p<0.05; |d|>1.1), whereas the remaining features did not differ significantly (p>0.30). Panels (**a**–**c**): HRV-derived features based on ln(RMSSD). Panels (**d**–**f**): HR-derived features. Panels (**g**,**h**): Demographic covariates (age and BMI). Box boundaries represent the interquartile range (IQR), the center line indicates the median, and whiskers extend to 1.5×IQR. *p*-values are reported without correction for multiple comparisons and should be interpreted as exploratory. An asterisk (*) indicates a statistically significant between-group difference (p<0.05). HRV, heart rate variability; HR, heart rate; SD, standard deviation; BMI, body mass index.

To assess effect sizes independently of the LASSO-based feature screening procedure, we performed univariate between-group comparisons at the subject level (Figure 3).

Two HRV dynamic indices exhibited statistically significant between-group differences with large effect sizes. Specifically, the nocturnal trend of the within-window standard deviation of ln(RMSSD), $b_{\sigma }^{\left(\ln(\mathrm{RMSSD})\right)}$, differed significantly between groups (p<0.05, Cohen’s d=−1.22). Similarly, the nocturnal trend of the within-window variance of ln(RMSSD), $b_{s^{2}}^{\left(\ln(\mathrm{RMSSD})\right)}$, also showed a significant difference (p<0.05, Cohen’s d=−1.17). In both cases, the magnitude of the effect sizes exceeded conventional thresholds for large effects.

### 3.4. Summary of Key Findings

Table 2 summarizes the candidate features identified through LASSO screening and their between-group comparison results.

The direction of these effects was consistent across indices. Participants in the lower-glycemic-risk group exhibited negative nocturnal trends (b<0), indicating that HRV variability decreased over the course of the night. This pattern is consistent with progressive autonomic stabilization during sleep, particularly reflecting parasympathetic modulation. In contrast, participants in the higher-glycemic-risk group showed positive nocturnal trends (b>0), indicating increasing HRV variability overnight, a pattern suggestive of persistent autonomic instability or disrupted autonomic regulation during sleep.

By contrast, the remaining features—including HR-derived dynamic indices, the nightly mean of within-window slopes ${\bar{\beta }}_{1}^{\left(\ln(\mathrm{RMSSD})\right)}$, age, and BMI—did not differ significantly between groups at the subject level (all p>0.30).

## 4. Discussion

This exploratory study identified candidate sleep-derived HR/HRV features that may be associated with glucose metabolism status. The key finding is that dynamic autonomic indices—capturing overnight trends and variability patterns—showed stronger associations with nocturnal glucose than conventional static mean values. Two HRV dynamic features (the nocturnal trends $b_{\sigma }^{\left(\ln(\mathrm{RMSSD})\right)}$ and $b_{s^{2}}^{\left(\ln(\mathrm{RMSSD})\right)}$) differed significantly between glycemic status groups with large effect sizes (|d|>1.1), providing converging evidence for their potential physiological relevance.

### 4.1. Physiological Interpretation of Dynamic HR/HRV Features

The prominence of dynamic indices in our feature selection results aligns with growing recognition that temporal patterns of autonomic activity—not just absolute levels—carry information about metabolic health. Prior longitudinal evidence supports this interpretation: the Rotterdam Study reported that changes in HRV trajectories over time predicted incident type 2 diabetes [14], and ELSA-Brasil found that unfavorable HRV patterns independently predicted diabetes risk [15].

A static mean HRV (e.g., ${\bar{\mu }}^{\left(\ln(\mathrm{RMSSD})\right)}$) reflects the overall level of parasympathetic activity. In contrast, the nocturnal trend of HRV variability ($b_{\sigma }^{\left(\ln(\mathrm{RMSSD})\right)}$) captures whether autonomic regulation stabilizes or remains unstable as sleep progresses. This distinction may be clinically meaningful: healthy sleep is characterized by progressive autonomic stabilization, particularly during deep non-rapid eye movement (NREM) sleep, whereas disrupted sleep architecture may manifest as persistent variability.

### 4.2. Interpretation of the Observed Directional Pattern

The directional pattern observed in this study—namely, a negative nocturnal trend in HRV variability ($b_{\sigma }^{\left(\ln(\mathrm{RMSSD})\right)}<0$) in the lower-glycemic-risk group contrasted with a positive trend ($b_{\sigma }^{\left(\ln(\mathrm{RMSSD})\right)}>0$) in the higher-glycemic-risk group—may be interpreted in light of several plausible physiological mechanisms.

Sleep architecture disruption may play an important role in the observed patterns. NREM sleep is generally characterized by parasympathetic dominance and relatively stable HRV, whereas rapid eye movement (REM) sleep shows greater sympathetic influence [44]. In healthy individuals, the predominance of deep NREM sleep early in the night may lead to progressive autonomic stabilization (b<0). Disruptions in sleep-stage continuity—commonly observed in metabolic dysfunction—could attenuate this stabilization pattern, resulting in b≈0 or b>0.

Additionally, micro-arousals and sleep fragmentation warrant consideration as potential contributors to the directional differences between groups. Experimental studies have shown that sleep fragmentation shifts autonomic balance toward sympathetic dominance and reduces insulin sensitivity [17]. The increasing HRV variability (b>0) observed in the higher-glycemic-risk group may reflect more frequent micro-arousals or disrupted sleep transitions, which transiently perturb autonomic regulation and increase within-window HRV dispersion.

Circadian autonomic dysregulation may also contribute to the observed patterns, potentially interacting with the mechanisms described above. Prior reviews have summarized links between sleep disruption, autonomic dysfunction, and insulin resistance [45,46]. The nocturnal trend of HRV variability may integrate these interacting processes into a single quantitative marker that captures the overall quality of overnight autonomic regulation.

### 4.3. Consistency with Prior Cohort Studies

The present findings are consistent with evidence from larger population-based cohorts. The Maastricht Study (N=2107) reported significantly lower HRV indices in individuals with prediabetes and type 2 diabetes [13]. In addition, Cheng et al. reported that sleep-time HRV measures were more strongly correlated with metabolic markers than waking-period measures [20]. Although the present study is smaller in scale, this consistency supports the rationale for focusing on sleep-time HR/HRV dynamics as candidate correlates of glycemic status.

### 4.4. Why Dynamic Features Were Selected over Static Means

The LASSO-based screening procedure preferentially selected dynamic indices—such as nocturnal trends and nightly variability of within-window slopes and speeds—over static mean measures. This pattern may reflect several complementary considerations. First, absolute HRV levels vary substantially between individuals due to factors that are not necessarily related to glycemic status, including physical fitness, resting heart rate, and genetic influences. In contrast, overnight trends may be less sensitive to these baseline differences, potentially providing a more direct window into physiological regulation linked to metabolic status.

Second, static mean values compress an entire sleep period into a single summary statistic and therefore discard information about temporal evolution. Dynamic indices preserve how autonomic activity changes across the night, which may be more tightly coupled to metabolic processes that also vary over the sleep cycle, including state-dependent shifts in autonomic balance and sleep-stage transitions.

Finally, these observations are consistent with theoretical perspectives on autonomic–metabolic interactions, which emphasize autonomic flexibility and the capacity to regulate appropriately across different physiological states [47]. Dynamic indices such as $b_{\sigma }^{\left(\ln(\mathrm{RMSSD})\right)}$ may better reflect this regulatory capacity than static averages, thereby offering greater sensitivity to subtle dysregulation.

Furthermore, the concept that dynamic variability patterns contain information beyond static averages has been observed in other modalities. For example, Piersanti et al. (2025) demonstrated that variability metrics of wrist skin temperature, rather than mean skin temperature, serve as novel digital biomarkers of glycemia [48]. This parallel finding suggests that metabolic dysregulation may manifest as subtle instability across multiple physiological systems, reinforcing the value of focusing on dynamic features in wearable-based assessments.

### 4.5. Limitations

This study has several important limitations that constrain interpretation of the findings. The sample size was small, with only 18 participants, which limits statistical power and precludes definitive inference. Accordingly, the identified associations should be regarded as preliminary signals rather than confirmatory evidence. In line with this exploratory aim, *p*-values were reported without correction for multiple comparisons and should be interpreted with appropriate caution.

The use of LASSO-based feature selection in a small-sample setting also introduces potential instability. When the number of predictors is large relative to the sample size, LASSO coefficients may be sensitive to data partitioning, and the specific features selected can vary across resamples. Approaches such as stability selection or bootstrap-based validation would strengthen confidence in the robustness of the identified candidate features.

In addition, this study employed a single-cohort design based on a convenience sample drawn from a Japanese population. As a result, the generalizability of the findings to other populations, age ranges, or ethnic groups remains uncertain, and external validation in independent cohorts is essential before broader conclusions can be drawn.

Glucose metabolism status was operationalized using CGM-derived eHbA1c rather than laboratory-measured HbA1c. Although the ADAG relationship linking mean glucose to HbA1c is well validated at the population level [35], individual-level discrepancies may arise due to biological variability in glycation kinetics and red blood cell lifespan. Future studies should incorporate laboratory HbA1c measurements as the reference standard. It should also be noted that both groups fell within a relatively narrow range of estimated HbA1c values near the prediabetes threshold. Such range restriction may attenuate observable between-group differences, and the identified associations should therefore be interpreted as hypothesis-generating rather than definitive.

Additionally, sex distribution differed marginally between groups (p=0.050), with a higher proportion of males in the higher glycemic risk group (6/7) compared with the lower glycemic risk group (3/11). Given known sex differences in both HRV and glucose metabolism, this imbalance represents a potential confound. Future studies should aim for sex-balanced recruitment or include sex as a covariate in multivariable models.

Finally, HR and HRV features were derived from consumer-grade wearable devices using photoplethysmography (PPG), which has known limitations compared with electrocardiography (ECG)-based measurements [23]. Although focusing on sleep periods and time-domain indices, such as RMSSD, may mitigate some sources of noise, measurement constraints inherent to consumer wearables should be considered when interpreting the results.

Furthermore, the sleep periods analyzed in this study were identified using the proprietary sleep-staging algorithm implemented in the Fitbit device. Although validation studies of earlier Fitbit models with sleep-staging capabilities have reported high sensitivity (0.95–0.96) for detecting sleep epochs against polysomnography [34], specificity for distinguishing quiet wakefulness from sleep has been shown to range from approximately 0.58 to 0.69 in these validation studies [34]. As a result, some epochs classified as sleep may include brief periods of wakefulness. Such misclassification could introduce noise into sleep-stage-agnostic HR/HRV estimates, although this effect is expected to be attenuated by our use of multi-night aggregation and by focusing on relative nocturnal trends rather than absolute values.

### 4.6. Future Directions

The exploratory findings of this study highlight several avenues for future confirmatory research. First, external validation in larger and independent cohorts represents a critical next step. Replication using samples of at least 50–100 participants, ideally with laboratory-measured HbA1c, will be necessary to evaluate the robustness and generalizability of the identified associations.

Second, the application of stability selection approaches may strengthen confidence in feature identification. For example, running LASSO-based screening across multiple bootstrap subsamples and quantifying selection frequencies would allow for assessment of the robustness of candidate features against sampling variability.

Third, longitudinal follow-up studies are needed to clarify the prognostic value of the proposed dynamic autonomic indices. Prospective designs examining whether these features predict future progression toward impaired glucose metabolism would help distinguish markers of current glycemic status from early indicators of disease development.

Finally, mechanistic investigations integrating wearable-derived autonomic measures with polysomnography could provide insight into the physiological substrates underlying the observed patterns. Such studies may clarify whether nocturnal HRV variability trends are related to specific aspects of sleep architecture, such as micro-arousal frequency or slow-wave sleep duration.

### 4.7. Night-to-Night Variability and Clinical Implications

Night-to-night variability is a critical consideration for any wearable-based screening approach, as sleep-time autonomic dynamics are influenced by multiple time-varying factors including sleep architecture (which varies naturally from night to night), prior-day physical activity, dietary intake, psychological stress, circadian phase, and environmental conditions. We acknowledge that substantial within-subject night-to-night variability exists in the extracted HR/HRV features.

The implications differ depending on whether assessment is based on single nights or multi-night aggregation. For individual-night classification, high variability would limit reliability. A single-night assessment might yield “low risk” one night and “high risk” another due to transient factors unrelated to underlying glycemic status. We explicitly caution that single-night assessments should not be used for definitive risk stratification. In contrast, for multi-night aggregation, averaging features across multiple nights (as we did at the participant level, approximately 10.5 nights per participant) attenuates transient noise and reveals more stable, participant-level autonomic patterns. This is analogous to averaging multiple blood pressure or fasting glucose measurements to improve diagnostic accuracy. Our between-group comparisons using participant-level aggregated features showed large effect sizes (Cohen’s |d|>1.1), suggesting that the signal-to-noise ratio improves substantially with multi-night averaging.

Emerging evidence suggests that intra-individual variability itself may carry prognostic information. In cardiovascular epidemiology, visit-to-visit variability in blood pressure predicts adverse outcomes independently of mean levels. By analogy, the magnitude of night-to-night fluctuations in autonomic features could reflect autonomic instability or impaired regulatory capacity linked to metabolic dysfunction.

We hypothesize that the dynamic trend features proposed in this study may be more robust to night-to-night variability than conventional static mean values. Static means are highly sensitive to absolute levels, which can vary substantially with prior-day activity or stress. In contrast, dynamic trends capture the “shape” of overnight recovery—the direction and pattern of autonomic change—which may reflect more fundamental regulatory processes that are less affected by transient perturbations.

For practical implementation, we propose a two-stage screening workflow: (1) initial screening via multi-night wearable-based assessment (aggregating data over 7–14 nights) to identify at-risk individuals; (2) confirmatory laboratory testing (measured HbA1c and/or oral glucose tolerance test) for those flagged as screen-positive. This approach leverages the scalability of consumer devices while maintaining diagnostic rigor.

Finally, we note that the “first-night effect” commonly observed in laboratory polysomnography is substantially mitigated by wearable monitoring in free-living conditions. Furthermore, our protocol explicitly excluded Day 1 as an acclimation period, ensuring that analyzed nights reflect habitual sleep patterns.

Future studies should (i) determine the optimal aggregation period (minimum number of nights required for stable feature estimates), (ii) assess whether within-subject variability metrics provide incremental predictive value beyond mean feature levels, and (iii) establish the test-retest reliability of dynamic features over longitudinal periods.

## 5. Conclusions

In this study using consumer wearables and CGM under free-living conditions, we examined whether sleep-derived autonomic signatures are associated with glucose metabolism status. By extracting window-level and night-level HR/HRV descriptors and applying Elastic Net feature screening, we found that dynamic autonomic indices—capturing overnight trends and changes in variability—were more informative than conventional static mean measures for explaining nocturnal mean glucose.

Among the candidate features, the nocturnal trends of within-window HRV variability quantified from ln(RMSSD) emerged as the most salient. Specifically, $b_{\sigma }^{\left(\ln(\mathrm{RMSSD})\right)}$ (the nocturnal trend of within-window standard deviation) and $b_{s^{2}}^{\left(\ln(\mathrm{RMSSD})\right)}$ (the nocturnal trend of within-window variance) not only retained non-zero coefficients during feature screening but also differed significantly between the lower- and higher-glycemic-risk groups with large effect sizes (both p<0.05; Cohen’s |d|>1.1). The directional pattern was consistent across both indices: HRV variability tended to decrease overnight in the lower-glycemic-risk group (b<0), whereas it tended to increase in the higher-glycemic-risk group (b>0). This contrast is physiologically plausible and aligns with prior evidence linking autonomic dysregulation and sleep-related instability to impaired glucose metabolism.

These results should be interpreted as hypothesis-generating given the small sample size, the single-cohort design, and the use of CGM-derived eHbA1c as an HbA1c-equivalent proxy. Nevertheless, the present findings suggest that sleep-time patterns of autonomic regulation—particularly nocturnal trends in HRV variability—may provide a practical, non-invasive set of candidate markers for screening-oriented assessment of glycemic risk using widely available consumer devices. Confirmatory studies in larger and independent cohorts, incorporating laboratory-measured HbA1c (and, where feasible, polysomnography or validated sleep metrics), are needed to evaluate generalizability, refine feature definitions, and determine whether these dynamic indices provide prognostic information for future progression to impaired glucose metabolism.

## Figures and Tables

**Table 1:** Participant characteristics by glycemic risk group.

Characteristic	Overall	Lower-Glycemic-Risk Group	Higher-Glycemic-Risk Group	*p* Value
*N* (participants)	18	11	7	—
Age (years)	39.6 ± 10.4	39.6 ± 4.5	39.4 ± 16.6	0.975
Sex (male/female)	9/9	3/8	6/1	0.050
BMI (kg/m^2^)	22.7 ± 2.3	22.8 ± 1.7	22.6 ± 3.2	0.832
Mean glucose (mg/dL)	112.3 ± 12.0	105.6 ± 3.8	122.8 ± 13.2	<0.001 ***
eHbA1c (%)	5.5 ± 0.4	5.3 ± 0.1	5.9 ± 0.5	<0.001 ***

Data are presented as mean ± SD. Lower-glycemic-risk group: eHbA1c < 5.5%; higher-glycemic-risk group: eHbA1c ≥ 5.5%. *** p<0.001.

**Table 2:** Summary of candidate dynamic features identified by LASSO screening. All listed features retained non-zero coefficients in the Elastic Net model (α=0.5; 5-fold CV).

Feature (Mathematical Notation)	Type	*p* ^†^	Cohen’s *d*
$b_{\sigma }^{\left(\ln(\mathrm{RMSSD})\right)}$: Nocturnal trend of $\sigma_{w}^{\left(\ln(\mathrm{RMSSD})\right)}$	HRV	0.044 *	−1.22
$b_{s^{2}}^{\left(\ln(\mathrm{RMSSD})\right)}$: Nocturnal trend of ${\left(s_{w}^{2}\right)}^{\left(\ln(\mathrm{RMSSD})\right)}$	HRV	0.044 *	−1.17
${\bar{\beta }}_{1}^{\left(\ln(\mathrm{RMSSD})\right)}$: Nightly mean of $\beta_{1,w}^{\left(\ln(\mathrm{RMSSD})\right)}$	HRV	>0.30	—
$b_{s^{2}}^{\left(\mathrm{HR}\right)}$: Nocturnal trend of ${\left(s_{w}^{2}\right)}^{\left(\mathrm{HR}\right)}$	HR	>0.30	—
${\bar{s_{w}^{2}}}^{\left(\mathrm{HR}\right)}$: Nightly mean of ${\left(s_{w}^{2}\right)}^{\left(\mathrm{HR}\right)}$	HR	>0.30	—
$\mathrm{SD}\;\left(v_{w}^{\left(\mathrm{HR}\right)}\right)$: Nightly SD of speed	HR	>0.30	—
Age	Demo	>0.30	—
BMI	Demo	>0.30	—

^†^ Between-group comparison (Welch’s *t*-test) at subject level. * p<0.05 (uncorrected). Notation: $\sigma_{w}$ = within-window SD; $s_{w}^{2}$ = within-window variance; $\beta_{1,w}$ = within-window slope; $v_{w}$ = within-window speed; *b* = nocturnal trend coefficient from Equation (3).

## Data Availability

Data is unavailable due to privacy or ethical restrictions.

## Supplementary Materials

The following supporting information can be downloaded at https://www.mdpi.com/article/10.3390/s26041118/s1: Table S1: Stage 1 (window-level): Definitions of HR- and HRV-derived features computed within each non-overlapping 30 min sleep window *w*; Table S2: Stage 2 (night-level): Definitions of night-level HR/HRV features obtained by aggregating Stage-1 window-level descriptors across windows.

## Abbreviations

The following abbreviations are used in this manuscript:

ANS	Autonomic nervous system
BMI	Body mass index
CAN	Cardiac autonomic neuropathy
CGM	Continuous glucose monitoring
CI	Confidence interval
DPP	Diabetes Prevention Program
ECG	Electrocardiography
eHbA1c	Estimated HbA1c
FPG	Fasting plasma glucose
GMI	Glucose management indicator
GV	Glycemic variability
HR	Heart rate
HRV	Heart rate variability
IDF	International Diabetes Federation
LASSO	Least Absolute Shrinkage and Selection Operator
lnRMSSD	Natural-log transformed RMSSD
REM	Rapid eye movement (sleep)
NREM	Non-rapid eye movement (sleep)
OGTT	Oral glucose tolerance test
PPG	Photoplethysmography
PRV	Pulse rate variability
RBF	Radial basis function
RMSSD	Root mean square of successive differences
SD	Standard deviation
SDNN	Standard deviation of NN intervals
TIR	Time in range
