# Supplementary material for: Dynamic Sleep-Derived Heart Rate and Heart Rate Variability Features Associated with Glucose Metabolism Status: An Exploratory Feature-Selection Study Using Consumer Wearables
Source: Sensors (Basel). 2026 Feb 9;26(4):1118. doi: 10.3390/s26041118 (PMC12944498; doi:10.3390/s26041118)
Supplement: Supplementary file 1 [file sensors-26-01118-s001.zip › Table S1.pdf]

Table S1. Stage 1 (window-level): Definitions of HR- and HRV-derived features computed within each non-overlapping 30 min sleep window w.

Mathematical definition	Unit	Description
HRV-derived (from $\ln \mathrm{RMSSD}$ *samples within window w)*		
$\mu_w^{(\ln \mathrm{RMSSD})} = \frac{1}{n_w} \sum_{i=1}^{n_w} \ln(\mathrm{RMSSD}_i)$	ln(ms)	Mean of ln(RMSSD) within window
$\sigma_w^{(\ln \mathrm{RMSSD})}$	ln(ms)	SD of ln(RMSSD) within window
$(\sigma_w^{(\ln \mathrm{RMSSD})})^2$	$(\ln(\mathrm{ms}))^2$	Variance of ln(RMSSD) within window
$\max_i \ln(\mathrm{RMSSD}_i)$	ln(ms)	Maximum ln(RMSSD) within window
$\min_i \ln(\mathrm{RMSSD}_i)$	ln(ms)	Minimum ln(RMSSD) within window
$\beta_{1,w}^{(\ln \mathrm{RMSSD})}$ from $\ln \mathrm{RMSSD} = \beta_0 + \beta_1 \cdot t_{\mathrm{hr}} + \varepsilon$	ln(ms)/h	Linear slope within window (time in hours)
HR-derived (from heart rate samples within window w)		
$\mu_w^{(\mathrm{HR})}$	bpm	Mean HR within window
$\sigma_w^{(\mathrm{HR})}$	bpm	SD of HR within window
$(\sigma_w^{(\mathrm{HR})})^2$	bpm^2	Variance of HR within window
$\max_i \mathrm{HR}_i$	bpm	Maximum HR within window
$\min_i \mathrm{HR}_i$	bpm	Minimum HR within window
$\beta_{1,w}^{(\mathrm{HR})}$ from $\mathrm{HR} = \beta_0 + \beta_1 \cdot t_{\mathrm{hr}} + \varepsilon$	bpm/h	Linear slope within window (time in hours)
$\frac{\mathrm{HR}(t_w + 30\,\mathrm{min}) - \mathrm{HR}(t_w)}{30\,\mathrm{min}}$	bpm/min	Endpoint-to-endpoint change rate

Notes: n_w: number of samples within window w. t_{hr}: elapsed time in hours from window start (range 0–0.5 h). ln(RMSSD) denotes the natural logarithm of RMSSD estimated from PPG-derived inter-beat intervals. Within-window slopes ($\beta_{1,w}$) are computed using elapsed time expressed in hours and therefore have units per hour (e.g., bpm/h for HR and ln(ms)/h for ln(RMSSD)).
