# Supplementary material for: Dynamic Sleep-Derived Heart Rate and Heart Rate Variability Features Associated with Glucose Metabolism Status: An Exploratory Feature-Selection Study Using Consumer Wearables
Source: Sensors (Basel). 2026 Feb 9;26(4):1118. doi: 10.3390/s26041118 (PMC12944498; doi:10.3390/s26041118)
Supplement: Supplementary file 1 [file sensors-26-01118-s001.zip › Table S2.pdf]

Table S2. Stage 2 (night-level): Definitions of night-level HR/HRV features obtained by aggregating Stage-1 window-level descriptors across windows.

Mathematical definition	Unit	Description
HRV-derived night-level features		
$\frac{1}{W_n}\sum_{w=1}^{W_n} \mu_w^{(\ln \mathrm{RMSSD})}$	ln(ms)	Nightly mean of window-level means
b from $\mu_w^{(\ln \mathrm{RMSSD})} = a + b \cdot t_{\mathrm{min},w} + \varepsilon$	ln(ms)/min	Nocturnal trend of window means
$\frac{1}{W_n}\sum_{w=1}^{W_n} \sigma_w^{(\ln \mathrm{RMSSD})}$	ln(ms)	Nightly mean of window-level SDs
b from $\sigma_w^{(\ln \mathrm{RMSSD})} = a + b \cdot t_{\mathrm{min},w} + \varepsilon$	ln(ms)/min	Nocturnal trend of window SDs
$\frac{1}{W_n}\sum_{w=1}^{W_n} (\sigma_w^{(\ln \mathrm{RMSSD})})^2$	$(\ln(\mathrm{ms}))^2$	Nightly mean of window variances
b from $(\sigma_w^{(\ln \mathrm{RMSSD})})^2 = a + b \cdot t_{\mathrm{min},w} + \varepsilon$	$(\ln(\mathrm{ms}))^2/\mathrm{min}$	Nocturnal trend of window variances
$\frac{1}{W_n}\sum_{w=1}^{W_n} \beta_{1,w}^{(\ln \mathrm{RMSSD})}$	ln(ms)/h	Nightly mean of within-window slopes
$\mathrm{SD}\{\beta_{1,w}^{(\ln \mathrm{RMSSD})}\}_{w=1}^{W_n}$	ln(ms)/h	Nightly SD of within-window slopes
HR-derived night-level features		
$\frac{1}{W_n}\sum_{w=1}^{W_n} \mu_w^{(\mathrm{HR})}$	bpm	Nightly mean of window-level means
b from $\mu_w^{(\mathrm{HR})} = a + b \cdot t_{\mathrm{min},w} + \varepsilon$	bpm/min	Nocturnal trend of window means
$\frac{1}{W_n}\sum_{w=1}^{W_n} \sigma_w^{(\mathrm{HR})}$	bpm	Nightly mean of window-level SDs
b from $\sigma_w^{(\mathrm{HR})} = a + b \cdot t_{\mathrm{min},w} + \varepsilon$	bpm/min	Nocturnal trend of window SDs
$\frac{1}{W_n}\sum_{w=1}^{W_n} (\sigma_w^{(\mathrm{HR})})^2$	bpm^2	Nightly mean of window variances
b from $(\sigma_w^{(\mathrm{HR})})^2 = a + b \cdot t_{\mathrm{min},w} + \varepsilon$	$\mathrm{bpm}^2/\mathrm{min}$	Nocturnal trend of window variances
$\frac{1}{W_n}\sum_{w=1}^{W_n} \beta_{1,w}^{(\mathrm{HR})}$	bpm/h	Nightly mean of within-window slopes
$\mathrm{SD}\{\beta_{1,w}^{(\mathrm{HR})}\}_{w=1}^{W_n}$	bpm/h	Nightly SD of within-window slopes
$\frac{1}{W_n}\sum_{w=1}^{W_n} \mathrm{speed}_w$	bpm/min	Nightly mean of window speeds
$\mathrm{SD}\{\mathrm{speed}_w\}_{w=1}^{W_n}$	bpm/min	Nightly SD of window speeds

Notes: W_n: number of valid 30 min sleep windows in night n. $t_{\mathrm{min},w}$: elapsed time in minutes from the first window of the night (used for nocturnal trends). Nocturnal trend slopes (b) are computed using elapsed time expressed in minutes and therefore have units per minute (e.g., bpm/min for HR and ln(ms)/min for ln(RMSSD)). This differs from within-window slopes ($\beta_{1,w}$), which use elapsed time in hours and thus have units per hour.
